# Supplementary material for: Digital Health Platforms in Saudi Arabia: Determinants from the COVID-19 Pandemic Experience
Source: Healthcare (Basel). 2021 Nov 8;9(11):1517. doi: 10.3390/healthcare9111517 (PMC8618772; doi:10.3390/healthcare9111517)
Supplement: Supplementary file 1 [file healthcare-09-01517-s001.zip › healthcare-1409845 supplementary to conversion.pdf]

Supplementary

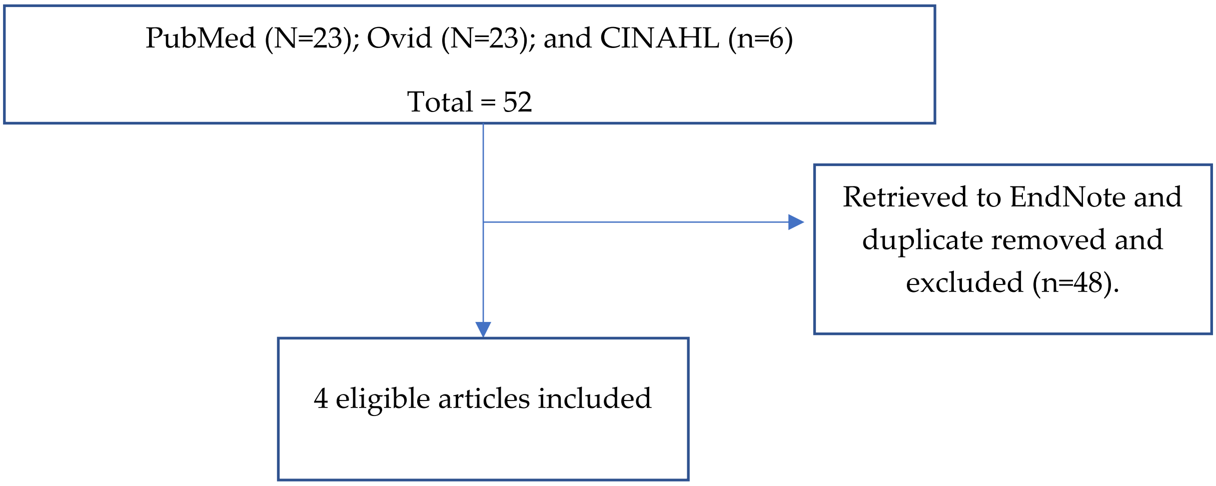

Figure S1. Inclusion and exclusion criteria.

Table S1. Eligibility criteria.

Inclusion and exclusion reasons	References
Critiquing digital health experience	[1-4]
Usability, knowledge d and experiences	[5-19]
Review	[20]

Supplementary Example of search strategy from one database

PubMed – up to September 2021

1. "COVID-19"[All Fields] OR "COVID-19"[MeSH Terms] OR "COVID-19 Vaccines"[All Fields] OR "COVID-19 Vaccines"[MeSH Terms] OR "COVID-19 serotherapy"[All Fields] OR "COVID-19 Nucleic Acid Testing"[All Fields] OR "covid-19 nucleic acid testing"[MeSH Terms] OR "COVID-19 Serological Testing"[All Fields] OR "covid-19 serological testing"[MeSH Terms] OR "COVID-19 Testing"[All Fields] OR "covid-19 testing"[MeSH Terms] OR "SARS-CoV-2"[All Fields] OR "sars-cov-2"[MeSH Terms] OR "Severe Acute Respiratory Syndrome Coronavirus 2"[All Fields] OR "NCOV"[All Fields] OR "2019 NCOV"[All Fields] OR (("coronavirus"[MeSH Terms] OR "coronavirus"[All Fields] OR "COV"[All Fields])

2. "Digit Health"[Journal] OR ("digital"[All Fields] AND "health"[All Fields]) OR "digital health"[All Fields]

3. ("Digit Health"[Journal] OR ("digital"[All Fields] AND "health"[All Fields]) OR "digital health"[All Fields]) AND platforms[All Fields]

4. "telemedicine"[MeSH Terms] OR "telemedicine"[All Fields] OR "telehealth"[All Fields]

5. "Saudi arabia"[MeSH Terms] OR ("saudi"[All Fields] AND "arabia"[All Fields]) OR "saudi arabia"[All Fields]

Ovid MEDLINE(R) ALL <1946 to September 11, 2021>

1 Mobile Applications/ or Telemedicine/ 37163

2 Telemedicine/ 30491

3 telehealth.mp. or Telemedicine/ 34369

4 health technology.mp. or Biomedical Technology/ 14001

5 digital health platforms.mp. 14

6 1 or 2 or 3 or 4 or 5 54179

7 COVID-19.mp. or COVID-19/178892

8 Saudi Arabia.mp. or Saudi Arabia/ 24663

9 6 and 7 and 8 23

CINAHL Data base – September 2021

S9	S6 AND S7 AND S8	Expanders - Apply equivalent subjects<br>Search modes - Boolean/Phrase	Interface - EBSCOhost Research Databases<br>Search Screen - Basic Search<br>Database - CINAHL	6
S8	Saudi Arabia	Expanders - Apply equivalent subjects<br>Search modes - Boolean/Phrase	Interface - EBSCOhost Research Databases<br>Search Screen - Basic Search<br>Database - CINAHL	6,859
S7	covid-19 or coronavirus or 2019-ncov or sars-cov-2 or cov-19	Expanders - Apply equivalent subjects	Interface - EBSCOhost Research Databases<br>Search Screen - Basic Search<br>Database - CINAHL	73,072

		Search modes - Boolean/Phrase		
S6	S1 OR S2 OR S3 OR S4 OR S5	Expanders - Apply equivalent subjects<br>Search modes - Boolean/Phrase	Interface - EBSCOhost Research Databases<br>Search Screen - Basic Search<br>Database - CINAHL	49,568
S5	digital health platforms	Expanders - Apply equivalent subjects<br>Search modes - Boolean/Phrase	Interface - EBSCOhost Research Databases<br>Search Screen - Basic Search<br>Database - CINAHL	105
S4	health technology	Expanders - Apply equivalent subjects<br>Search modes - Boolean/Phrase	Interface - EBSCOhost Research Databases<br>Search Screen - Basic Search<br>Database - CINAHL	13,871
S3	telehealth	Expanders - Apply equivalent subjects<br>Search modes - Boolean/Phrase	Interface - EBSCOhost Research Databases<br>Search Screen - Basic Search<br>Database - CINAHL	22,885
S2	Telemedicine	Expanders - Apply equivalent subjects<br>Search modes - Boolean/Phrase	Interface - EBSCOhost Research Databases<br>Search Screen - Basic Search<br>Database - CINAHL	22,636
S1	Mobile Applications	Expanders - Apply equivalent subjects<br>Search modes - Boolean/Phrase	Interface - EBSCOhost Research Databases<br>Search Screen - Basic Search<br>Database - CINAHL	11,260

Supplementary Reference

1. Alghamdi, S.M., J.S. Alqahtani, and A.M. Aldhahir, *Current status of telehealth in Saudi Arabia during COVID-19.* Journal of Family and Community Medicine, 2020. **27**(3): p. 208-211.
2. Eddine, I.S. and H.S. Zedan, *Telehealth Role During the COVID-19 Pandemic: Lessons Learned from Health Care Providers in Saudi Arabia.* Telemedicine Journal & E Health, 2021. **13**: p. 13.
3. Hassounah, M., H. Raheel, and M. Alhefzi, *Digital Response During the COVID-19 Pandemic in Saudi Arabia.* Journal of Medical Internet Research, 2020. **22**(9): p. e19338.
4. Al Knawy, B., et al., *The Riyadh Declaration: the role of digital health in fighting pandemics.* The Lancet, 2020. **396**(10262): p. 1537-1539.
5. Abdel Nasser, A., et al., *Measuring the Patients' Satisfaction About Telemedicine Used in Saudi Arabia During COVID-19 Pandemic.* Cureus, 2021. **13**(2): p. e13382.
6. Aboalshamat, K.T., *Awareness of, Beliefs about, Practices of, and Barriers to Teledentistry among Dental Students and the Implications for Saudi Arabia Vision 2030 and Coronavirus Pandemic.* Journal of International Society of Preventive & Community Dentistry, 2020. **10**(4): p. 431-437.
7. Al-Rayes, S.A., et al., *Public Awareness and Utilization of 937-Telephone Health Services in the Kingdom of Saudi Arabia Before and During the COVID-19 Pandemic: Longitudinal Study.* Journal of Medical Internet Research, 2021. **23**(7): p. e27618.
8. AlHumaid, J., S. Ali, and I. Farooq, *The psychological effects of the COVID-19 pandemic and coping with them in Saudi Arabia.* Psychological Trauma:Theory, Pesearch, Practice and Policy, 2020. **12**(5): p. 505-507.
9. Almufarriji, R.M., A.O. Alobaid, and F.A. Alsubaie, *Saudi neurosurgery residency and COVID-19: How are we coping?* Neurosciences, 2020. **25**(5): p. 343-344.
10. Alnajashi, H. and R. Jabbad, *Behavioral practices of patients with multiple sclerosis during Covid-19 pandemic.* PLoS ONE [Electronic Resource], 2020. **15**(10): p. e0241103.
11. Alwabili, A.A., et al., *Measurement of Patient Satisfaction With the Trend of Virtual Clinics During the COVID-19 Pandemic.* Cureus, 2021. **13**(6): p. e16016.
12. Assiri, A.H., et al., *The Modality Preferred by Population of Health Services Provided During COVID-19 and Assessment of Satisfaction Among Adults in Kingdom of Saudi Arabia.* Cureus, 2021. **13**(7): p. e16190.
13. Karim, S.I., F. Irfan, and M.A. Batais, *Becoming virtual: a preliminary experience of outpatient primary care during COVID-19 pandemic.* The Pan African medical journal, 2020. **37**: p. 262.
14. Meo, S.A., *COVID-19 Pandemic: Saudi Arabia's Role at National and International Levels.* Journal of Diabetes Science & Technology, 2020. **14**(4): p. 758-759.
15. Yamin, M.A.Y. and B.A. Alyoubi, *Adoption of telemedicine applications among Saudi citizens during COVID-19 pandemic: An alternative health delivery system.* Journal of Infection and Public Health, 2020. **13**(12): p. 1845-1855.
16. Al-Sofiani, M.E., et al., *Rapid Implementation of a Diabetes Telemedicine Clinic During the Coronavirus Disease 2019 Outbreak: Our Protocol, Experience, and Satisfaction Reports in Saudi Arabia.* Journal of Diabetes Science & Technology, 2021. **15**(2): p. 329-338.
17. Alshaqaq, A., et al., *Coronavirus Disease 2019 and Kidney Transplantation in Saudi Arabia: Outcomes and Future Opportunities.* Annals of Transplantation, 2021. **26**: p. e931832.
18. Alshareef, M., et al., *Utilization of telemedicine in rhinologic practice during COVID-19 pandemic.* American Journal of Otolaryngology, 2021. **42**(3): p. 102929.
19. Magliah, S.F., et al., *Managing Type 1 Diabetes among Saudi adults on insulin pump therapy during the COVID-19 lockdown.* Diabetes & Metabolic Syndrome, 2021. **15**(1): p. 63-68.
20. Alanzi, T., *A Review of Mobile Applications Available in the App and Google Play Stores Used During the COVID-19 Outbreak.* Journal of multidisciplinary healthcare, 2021. **14**: p. 45-57.
